# Treatment of cam-type femoroacetabular impingement using anterolateral mini-open and arthroscopic osteochondroplasty

**DOI:** 10.1186/s13018-019-1257-z

**Published:** 2019-07-17

**Authors:** Cheng-Ta Wu, Mohammed Mahameed, Po-Chun Lin, Yu-Der Lu, Feng-Chi Kuo, Mel S. Lee

**Affiliations:** 1 Department of Orthopaedic Surgery, Kaohsiung Chang Gung Memorial Hospital, Kaohsiung, Taiwan; 2 Chang Gung University, College of Medicine, 123, Ta Pei Road, Niao Sung District, Kaohsiung, Taiwan

## Abstract

**Background:**

Femoroacetabular impingement (FAI) is associated with decreased hip function and early hip osteoarthritis. Surgical treatment is often required to halt the process of mechanical degeneration. The study investigated the short-to-midterm results of using a modified anterolateral mini-open and arthroscopic osteochondroplasty in the treatment of cam-type FAI.

**Methods:**

Thirty-six patients (39 hips), with the mean age of 43.6 years, who had cam-type FAI, were operated by a mini-open and arthroscopy-assisted osteochondroplasty via the Watson-Jones interval between 2002 and 2016. Radiographic parameters and Harris hip scores were retrospectively analyzed after a mean follow-up of 44 months.

**Results:**

Of the 39 hips, the mean Harris hip score significantly improved from 61.1 preoperatively to 84.2 postoperatively (*P* < 0.01). There were nine hips (23%) undergoing total hip arthroplasty (THA) at a mean of 22 months (range, 3~64 months) due to progression of hip osteoarthritis. The 5-year survival for hip preserving was 74.9%. Cox proportional-hazards model showed that age ≥ 55 years (*P* = 0.03) and preoperative Tönnis stage II (*P* = 0.02) were independent risk factors for conversion to THA.

**Conclusions:**

The mini-open and arthroscopic approach allowed direct visualization and improved quality of the osteochondroplasty of FAI hip while avoiding the need for surgical dislocation. This technique could be a safe and viable option for symptomatic cam-type FAI patients to relieve symptoms and improve hip function.

## Introduction

Femoroacetabular impingement (FAI) is one of the most common causes of groin pain in young adults [1]. The prevalence ranges around 10% to 15% in young active patients and up to 94% of young patients with hip pain [2]. Emerging evidence has shown that FAI is a major cause of acetabular labral and cartilage injuries, and is also recognized as a significant contributing factor in the development of early hip osteoarthritis [3, 4]. The underlying pathomechanism is an abnormal contact and shear force between the femoral head and the rim of acetabulum during physiological range of motion [5]. In cam-type lesion, the non-spherical, bumped anterosuperior femoral head-neck junction pressurized and squeezed the cartilage adjacent to the acetabular labrum repetitively during deep hip flexion. The pathological contact results in extensively significant articular cartilage damage first, followed by the labrum lesions, which is why pain often occurs later [6, 7]. In comparison, earlier hip pain could be noticed in pincer-type FAI because the labrum lesions resulted from the impingement of a retroverted or overcovered acetabular margin against the femoral neck are often antecedent and more dominant [7]. Therefore, a symptomatic cam-type FAI possibly warrants more clinical concern and prompt intervention to prevent deterioration of chondral and labral injuries, and the development of hip osteoarthritis.

The aims of surgery for FAI are to correct bony deformities and address associated labral or chondral lesions [8]. Three main surgical approaches currently being used are open surgical dislocation, hip arthroscopy, and minimally invasive open surgery, each with their advantages and disadvantages [9–11]. Although surgical hip dislocation and open osteochondroplasty has satisfactory clinical results and been considered the gold-standard procedure, complication rates related to intraarticular adhesion and trochanteric osteotomy were not uncommon [12, 13]. Arthroscopy has become increasingly popular in terms of less traumatic to soft tissues and rapid return to sport activity [14, 15]. However, femoral neurovascular injury, higher revision rate for inadequate symptoms relief, and incomplete bony correction remained a concern [16, 17]. The use of mini-open technique with arthroscopic assistance is less often reported in the literature [18]. The advantages of this approach include both minimal soft tissue invasion and improved quality of the osteochondroplasty by direct visualization on femoral head and neck that would otherwise only be obtained with surgical dislocation [19]. The assistance of arthroscopy provided detailed visualization and management of chondral and labral injuries in a small wound [20]. The technique has been shown to provide rapid recovery and adequate symptom relief to successfully treat FAI in athletic population [8].

The purpose of this retrospective study was to analyze the short-to-midterm results of using a modified anterolateral mini-open osteochondroplasty with arthroscopy-assistance in the treatment of cam-type FAI. We hypothesized that using this technique could ameliorate hip symptoms related to mechanical impingement and improve hip function. The progression of hip osteoarthritis and the need of subsequent THA were also evaluated. Clinical factors that may portend successful or unsuccessful surgical outcomes were investigated.

## Materials and methods

### Study design

From Jan 2002 to Dec 2016, we treated 43 hips in 40 patients with cam-type FAI using a modified anterolateral mini-open (Watson-Jones) approach and arthroscopy-assisted osteochondroplasty. The diagnosis of FAI was based on patient history and clinical impingement test, conventional anteroposterior (AP) pelvis, cross-table hip lateral radiographs, and MRI [6]. The indications for surgery and the inclusion criteria of this retrospectively designed study consisted of patients with (1) persistent activity-related groin pain after at least 3 months of non-operative treatment; (2) cam-type FAI, which was defined as an alpha angle exceeding 50° on axial view and/or a pistol grip deformity [21]; and (3) follow-up for at least 24 months or until an end-point occurred. Patients were precluded from this procedure if he or she had (1) advanced hip osteoarthritis, defined as Tönnis stage III [22]; (2) mixed or pincer type FAI, defined as a lateral center-edge (LCE) angle more than 33° and/or acetabular index (AI) less than 3° [21]; or (3) Legg-Calve′-Perthes disease. Based on our experience and the literature, patients with pincer impingement, marked protrusion of the femoral head, and pronounced acetabular retroversion were treated in favor of open surgical dislocation [23].

At the final visit, 39 hips in 36 patients were available for this retrospective review (four patients lost follow-up). Demographic data, intraoperative findings of labral and cartilage injuries, visual analogue scale (VAS), Harris hip score (HHS), and radiographic parameters including Tönnis angle, femoral head-neck offset, LCE angle, AI, and extrusion index were recorded in detail. Labral damage was classified according to Beck et al. [5]. Cartilage damage was classified according to the Outerbridge classification [24]. The study was conducted with a waiver of patient consent but approved by the Institution Review Board of the hospital.

### Surgical procedure

The patient was prepared in lateral decubitus position, and about a 4-cm incision was made between anterior superior iliac spine and greater trochanter. The hip joint was approach through the interval between tensor fascia lata and gluteus medius (the Watson-Jones interval). A capsulectomy was performed to expose the anterior and anterolateral aspect of femoral head-neck junction. Distraction of the affected limb was applied manually by assistants, and a 30° arthroscope was used to examine the acetabular chondral and labral integrity. The torn labrum was partially trimmed and debrided unless it was repairable. The chondral injury was mostly debrided with or without additional microfracture.

**Fig. 1:**
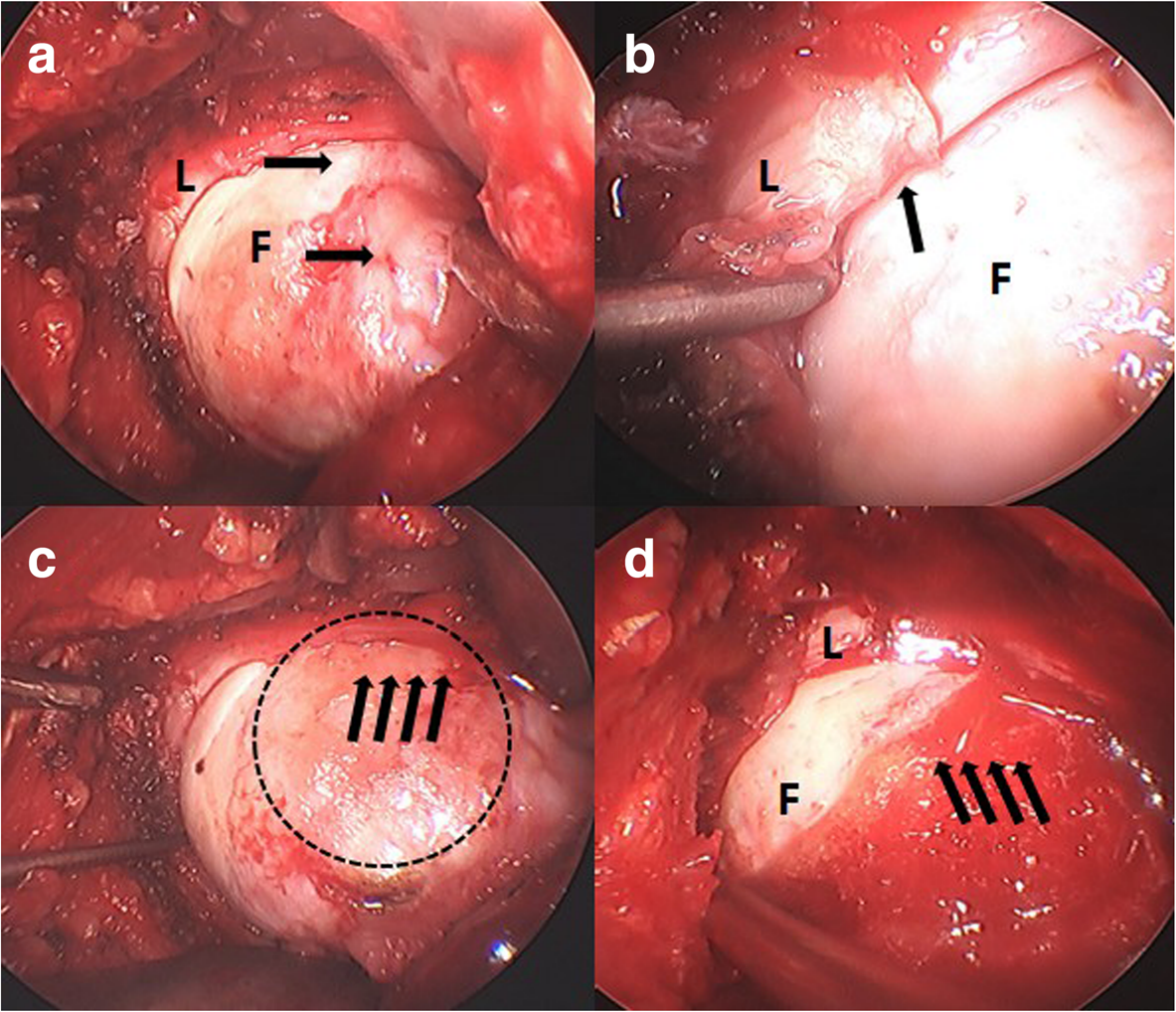
Intraoperative images under dry arthroscopy. **a** The integrity of the labrum (L) and the bony bumps (arrows) on the anterosuperior femoral head-neck junction (F) could be clearly examined and identified. **b** A degenerative labral lesion (arrows) resulted from repetitive cam-type impingement. **c** The pressurization and squeeze of bony bumps into labrum and cartilage could be reproduced by hip flexion and internal rotation; the dotted circle represented the possible osteochondroplasty site. **d** Restoration of femoral head-neck offset after osteochondroplasty (arrows)

The bony deformity causing the impingement could be identified by scope, direct vision, and putting the index finger in the junction during the impingement maneuver (Fig. 1). The anterior and anteroinferior portion of the femoral neck could be approached by hip flexion and external rotation, and the posterior and posterosuperior aspect by hip extension and internal rotation. High-speed diamond burr and small osteotome were used to remove the bony bump and restore the head-neck offset. Care should be taken to avoid excessive trimming of femoral neck, which might increase the risk of postoperative fracture. The femoroacetabular clearance was verified by scope, direct vision, and finger test to obtain an impingement-free range of motion, especially in hip flexion and internal rotation.

The patients were instructed to follow the postoperative protocol, including crutches use with partial weight bearing for 1~2 weeks after the surgery, and were advanced to full weight bearing thereafter. Low-impact activity could be resumed at postoperative 6~8 weeks, and high-impact activity at postoperative 12 weeks. The patients were followed up clinically and radiographically at 6 weeks, 3 months, 6 months, and 12 months postoperatively and then annually after the index surgery.

### Outcomes

At the last follow-up, patients were examined with a complete workup consisted of the history (symptom-free, conversion to THA, or any revision surgery), hip ROM and impingement test, and evaluation of VAS and HHS. Standardized pelvis AP and cross-table hip lateral radiographs were routinely taken to assess signs of osteoarthritis (Tönnis stage) [22], heterotopic ossification, pistol grip deformity, alpha angle, and regrowth of cam lesion.

The procedure was defined clinically successful if the patients were symptomatically and functionally improved (HHS ≥ 80) without the need for additional surgery and favorably preserved the native hip joint without conversion to THA. If the symptoms did not relieve completely (VAS > 3 and HHS < 80) and the radiographs showed inadequate correction of femoral head-neck offset or regrowth of cam lesions without signs of hip osteoarthritis (Tönnis stage 0 or I), revision osteochondroplasty by the same procedure would be advised. In contrast, if signs of hip osteoarthritis progressed to Tönnis stage II or III, THA would be considered. Patients with postoperative HHS < 80 at final visit, requiring any revision surgery or conversion to THA, were defined as clinical failure. Possible complications related to the surgical procedure, e.g., fracture of femoral neck or infection, were recorded if it happened.

### Statistical analysis

The χ^2^ test or Fisher’s exact test was used when analyzing the differences of categorical variables between patients of clinical success and clinical failure. Differences of VAS and HHS between patients of clinical success and clinical failure were compared by the Wilcoxon-Mann-Whitney test. Statistical differences in survival rate were compared using log-rank analysis of Kaplan-Meier survival curves with the end point of conversion to THA or any revision surgery. The Cox proportional hazards model was used to analyze the independent factors associated with clinical failure. All tests were two-sided, and *P* <  0.05 was considered significant. All statistical comparisons were made using the Statistical Package for Social Sciences (SPSS) (version 20; SPSS Inc., Chicago, Illinois).

## Results

**Fig. 2:**
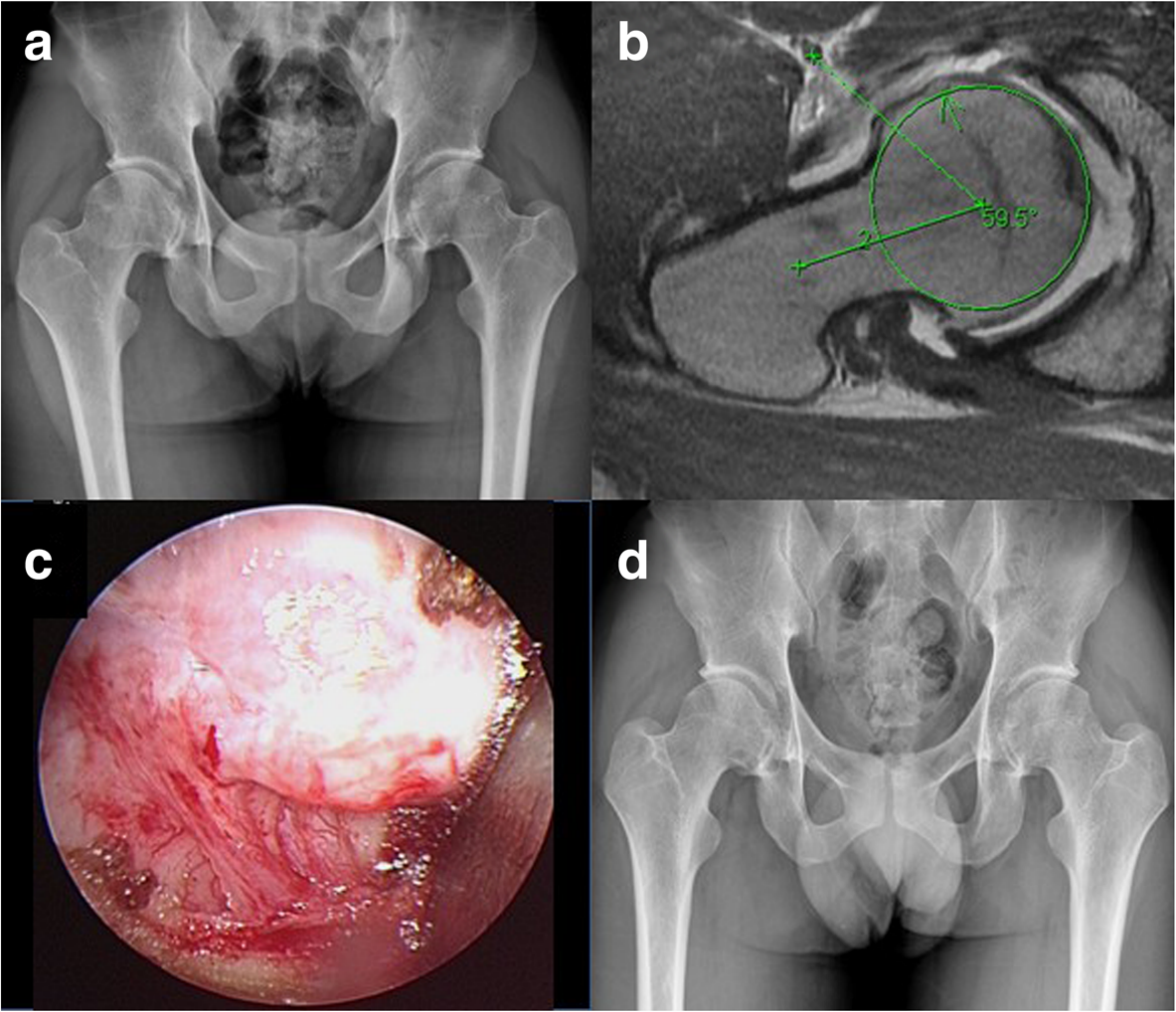
A 23-year-old male patient with bilateral proximal femur cam-type FAI. **a** Preoperative pelvis radiograph. **b** Preoperative right hip MRI axial view. **c** intraoperative arthroscopic image showing the bony bump. **d** Follow-up radiography of pelvis AP view at postoperative 3 years

**Fig. 3:**
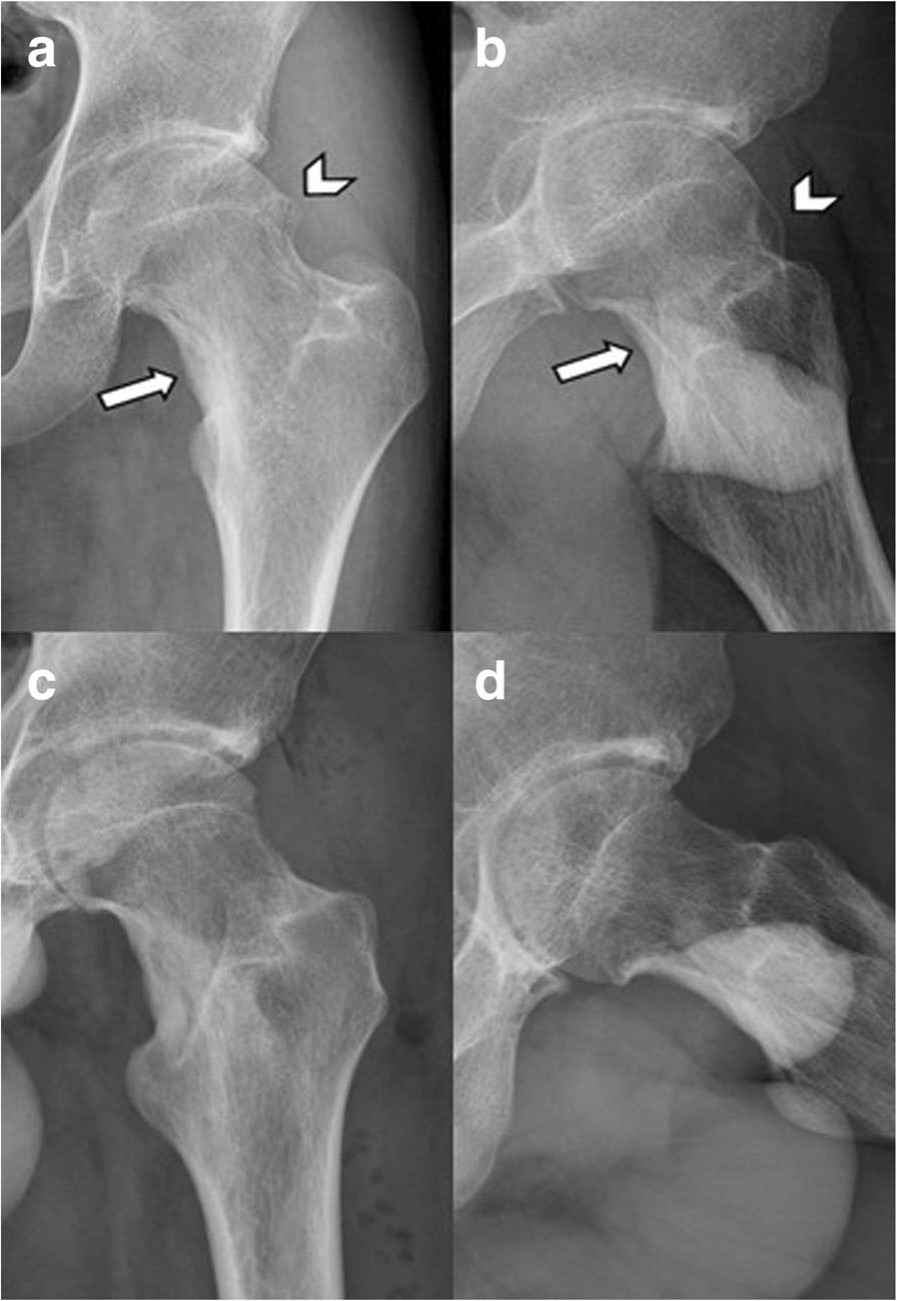
An 18-year-old male patient, who has undergone curettage and bone grafting for left femoral neck osteoid osteoma 2 years ago, presented with left proximal femur cam-type FAI. **a**, **b** Preoperative hip AP and lateral view showing the location of osteoid osteoma (arrow) and the bony bump (arrowhead). **c**, **d** Follow-up radiography at postoperative 2 years after complete removal of the cam lesion

The preoperative Tönnis stage of 39 hips available for evaluation were nine hips in stage 0, 15 hips stage I, and 15 hips stage II, respectively. The patients were 19~64 years of age (mean, 43.6 ± 14.0) at the time of operation. Three patients underwent bilateral surgeries (Fig. 2). Five cases had prior hips surgeries: two post-traumatic femoral head deformity due to previous hip fracture-dislocation; one prior hip arthroscopy for synovial chondromatosis; one with history of arthrotomy for septic hip at childhood; and one femoral neck osteoid osteotoma (Fig. 3). Labral tear was diagnosed in 21 hips by preoperative MRI and intraoperative findings. They were partially trimmed and excised for degenerative or unrepairable tear. The mean follow-up was 44 months (range, 3~180 months). There were no significant differences between patients of clinical success and clinical failure in terms of the proportion of patients with BMI ≥ 25 kg/m^2^, labral damage, and preoperative radiographic parameters. However, significant differences were noted with regard to gender, the proportion of patients with age ≥ 55 years, chondral damage, and preoperative Tönnis stage (Tables 1 and 2). The mean HHS of all hips improved from 61.1 points (range, 40~78 points) preoperatively to 84.2 points (range, 50~92 points) at the final follow-up. The improvement in HHS was significantly higher for hips without subsequent THA than those requiring subsequent THA (25.6 ± 11.5 vs. 14.4 ± 6.3, *P* = 0.001). There was no patient sustained femoral neck fracture, infection, heterotopic ossification, or paresthesia of lateral femoral cutaneous nerve after the surgery.

**Table 1:** Demographic and clinical characteristics of the study patients

	Total sample No.	Clinical success (*N* = 30)	Failure (THA, *N* = 9)	*P* value
Gender (M/F)	22/17	20/10	2/7	0.026
Mean age, years	43.6 ± 14.0	40.7 ± 13.4	53.2 ± 12.3	0.019
≥ 55 years/< 55 years	9/30	4/26	5/4	0.018
Mean BMI	24.5 ± 4.0	24.0 ± 3.8	26.1 ± 4.7	0.309
≥ 25/< 25	15/24	10 / 20	5 / 4	0.266
Side				
R/L	22/17	16/14	6/3	0.704
Labral damage				0.198
No tear	18	16	2	
Full-thickness	11	8	3	
Degenerative	10	6	4	
Cartilage damage				0.002
Gr 0	10	10	0	
Gr I	11	11	0	
Gr II	10	5	5	
Gr III~IV	8	4	4	
Pre-op Tönnis stage				0.021
Stage 0	9	9	0	
Stage I	15	13	2	
Stage II	15	8	7	
Mean symptoms duration (months)	33.6 ± 31.7	32.5 ± 31.1	37.3 ± 35.3	0.624
Spur regrowth	5/39	3/30	2/9	0.586
Pre-op VAS	6.8 ± 1.0	6.7 ± 1.1	7.1 ± 0.6	0.309
Post-op VAS	2.2 ± 2.1	1.3 ± 1.2	5.2 ± 1.1	< 0.001
VAS decrease	4.6 ± 2.1	5.4 ± 1.5	1.9 ± 1.4	< 0.001
Pre-op HHS	61.1 ± 9.3	62.5 ± 9.4	56.4 ± 7.8	0.068
Post-op HHS	84.2 ± 10.8	88.2 ± 8.8	70.9 ± 3.6	< 0.001
HHS increase	23.1 ± 11.5	25.6 ± 11.5	14.4 ± 6.3	0.001

**Table 2:** Radiographic parameters of the study patients

	Total sample No.	Clinical success (*N* = 30)	Failure (THA, *N* = 9)	*P* value
Pre-op Alpha angle	82.1 ± 13.0	81.2 ± 12.8	84.8 ± 14.0	0.505
Femoral offset	6.2 ± 2.8	6.4 ± 2.8	5.4 ± 2.9	0.229
Extrusion index	13.1 ± 5.7	13.4 ± 5.9	12.2 ± 5.1	0.700
Acetabular angle	39.2 ± 4.1	39.1 ± 3.8	39.7 ± 5.1	0.973
Acetabular index	9.4 ± 5.8	9.3 ± 6.1	9.9 ± 4.7	0.463
LCE angle	34.5 ± 7.9	34.8 ± 7.7	33.5 ± 8.7	0.731

### Conversion to THA

**Fig. 4:**
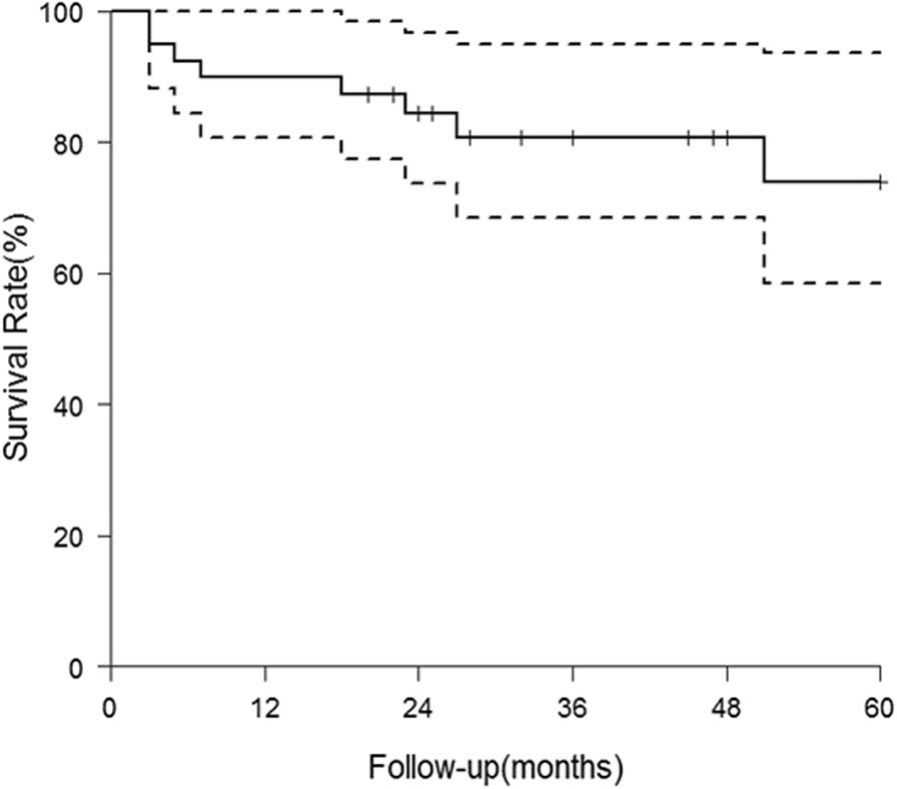
Survival curve of clinical failure with conversion to THA as endpoint

**Fig. 5:**
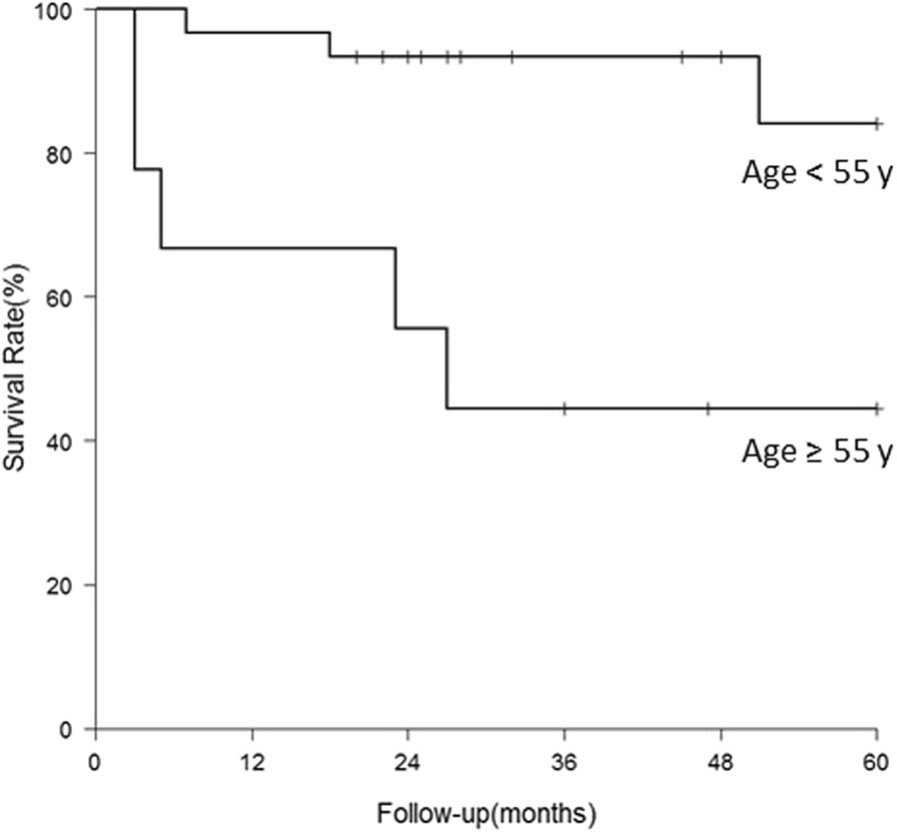
Survival curve of clinical failure by age

**Fig. 6:**
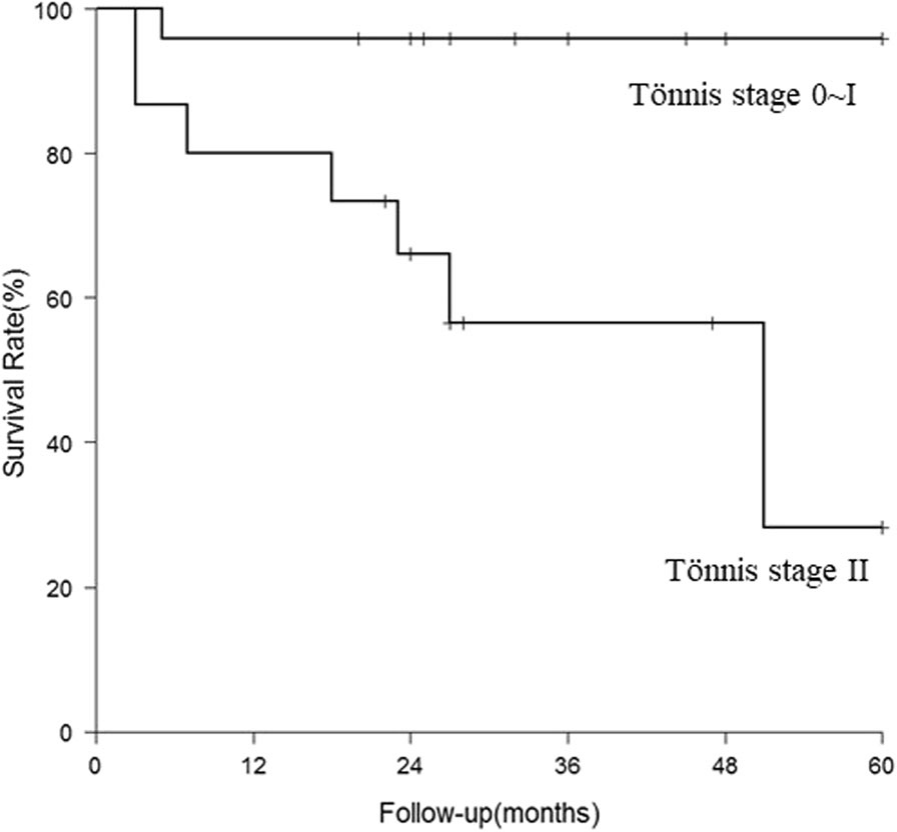
Survival curve of clinical failure by Tönnis stage

There was no patient undergoing revision procedure. Nine hips with a postoperative HHS < 80 ended up with THA at a mean of 22 months (range, 3~64 months) after the index procedure. Therefore, conversion to THA was used as the endpoint of clinical failure in the analysis. THAs were done via the same Watson-Jones approach along the previous scar. The 5-year survival for hip preserving was 74.9% (Fig. 4). Table 3 summarized the possible risk factors leading the hips to THA. Hips were more prone to failure in patients with age ≥ 55 years (*P* < 0.01), hips with Gr III~IV cartilage injury (*P* = 0.02), and preoperative Tönnis stage II (*P* < 0.01) based on the 5-year survivorship analysis. However, no significant difference was found in the survivorship when stratified by gender (*P* = 0.05), BMI ≥ 25 kg/m^2^ (*P* = 0.16), or the presence of labral tear (*P* = 0.33). Cox proportional hazards model showed that age ≥ 55 years (*P* = 0.03; hazard ratio [HR] 4.4; 95% CI 1.12–17.29) and preoperative degenerative Tönnis stage II (*P* = 0.02; HR 14.71; 95% CI 1.66–130.02) were independent risk factors for conversion to THA (Table 4). Significant differences in survivorship stratified by age and Tönnis stage were shown in Figs. 5 and 6.

**Table 3:** The analytic results and survival rate of hips conversion to THA

Index	Survival rate (3 years)	*P* value	Survival rate (5 years)	*P* value
Overall sample	0.806 (0.685, 0.949)		0.739 (0.584, 0.936)	
Gender		0.017		0.054
Male	0.955 (0.871, 1.000)		0.818 (0.597, 1.000)	
Female	0.623 (0.422, 0.920)		0.623 (0.422, 0.920)	
Age		0.001		0.002
≥ 55	0.444 (0.214, 0.923)		0.444 (0.214, 0.923)	
< 55	0.933 (0.848, 1.000)		0.840 (0.669, 1.000)	
BMI		0.268		0.162
≥ 25	0.727 (0.531, 0.996)		0.606 (0.376, 0.976)	
< 25	0.840 (0.682, 1.000)		0.840 (0.682, 1.000)	
Labral tear		0.353		0.332
Y	0.756 (0.590, 0.968)		0.687 (0.504, 0.937)	
N	0.881 (0.739, 1.000)		0.881 (0.739, 1.000)	
Cartilage injury		0.082		0.023
Gr 0~II	0.851 (0.723, 1.000)		0.851 (0.723, 1.000)	
Gr III~IV	0.625 (0.365, 1.000)		0.417 (0.159, 1.000)	
Tönnis stage		0.005		0.001
Stage 0~I	0.958 (0.882, 1.000)		0.958 (0.882, 1.000)	
Stage II	0.556 (0.351, 0.911)		0.283 (0.065, 1.000)

**Table 4:** The results of Cox proportional-hazards model for hips conversion to THA

Risk factors to clinical failure	Hazard ratio (95% CI)	*P* value	Adjusted hazard ratio (95% CI)	*P* value
Male vs. Female	0.201 (0.042, 0.968)	0.045		
Age, ≥ 55 vs. < 55	5.153 (1.378, 19.269)	0.015	4.399 (1.119, 17.292)	0.034
BMI, ≥ 25 vs. < 25	1.900 (0.505, 7.155)	0.343		
Labral tear		0.321		
Y vs. N	2.273 (0.449, 11.521)			
Cartilage injury		0.065		
Gr III~IV vs. Gr 0~II	3.467 (0.926, 12.978)			
Tönnis stage		0.011	14.706 (1.663, 130.023)	0.016
Stage II vs. Stage 0~I	15.423 (1.858, 128.018)

## Discussion

The surgical goals for FAI are to reshape the cam and/or pincer bony deformities, and to repair or debride the chondrolabral injuries [20, 23]. Surgical hip dislocation was conventionally considered as the gold-standard treatment since it allowed an unrestricted visualization of hip joint and provided wide access to treat the bony pathomorphologies [12, 25]. However, it was technically demanding and associated with variable rates of complications such as trochanteric nonunion or arthrofibrosis [13, 25]. In our study of using mini-open and arthroscopy-assisted technique, all the patients had an improvement in HHS and decrement in VAS regardless of clinical success or failure at the final follow-up. Seventy-seven percent of hips had satisfactory outcomes without the need for revision surgery or THA. We have also found that age ≥ 55 years and preoperative degenerative Tönnis stage II were independent risk factors for future THA conversion. With our considerable experience in THA via the modified Watson-Jones approach [26, 27], we were able to perform the osteochondroplasty on the head-neck junction with a small intermuscular interval, and use arthroscopic technique to manage the labral and cartilage injuries in patients with cam-type FAI.

Several studies have reported the results of using mini-open technique in the treatment of FAI [8, 9, 19]. Most of the authors used anterior Hueter approach on an extension table or fracture table, taking the advantage of intermuscular and internervous dissection [18]. We believe that both the mini-open Hueter anterior approach and our anterolateral Watson-Jones approach could be efficacious to treat the pure cam-type FAI cases. The most frequent complication regarding Hueter approach, however, was the potential injury to posterior branches of the lateral femoral cutaneous nerve, with incidences ranging from 0 to 20% [8, 19]. In the current study, the use of Watson-Jones interval between tensor fascia lata and gluteus medius could avoid this complication.

The use of mini-open technique with arthroscopy-assistance could be as efficacious as other approaches to correct the hip pathomorphologies and preserve native hip joint from replacement. Peters et al. reported 22% of patients undergoing THA at 32 months follow-up after hip surgical dislocation [28]. Beck et al. showed 26% of THA conversion rate at 56 months follow-up [12]. By using hip arthroscopy, 34% of patients underwent THA within 10 years in a prospective study conducted by Menge et al. [29], and about 20% of failure rate (defined as conversion to THA, progression of osteoarthritis, and poor clinical functional scores) was reported in another study at a mean of 7 years [21]. Our study showed a comparable conversion rate (23%) at a mean follow-up of 44 months (Table 5). While arthroscopic surgery for FAI is more favorable than the open dislocation in terms of general health-related quality of life [13, 14], pure hip arthroscopy needs excessive traction during surgery and is associated with the risk of neural damage [34]. In addition, the hip arthroscopy may result in inadequate resection of bony deformities, especially in complex cases or in the early stage of learning curve [16, 17, 34]. The most distal and posterior head-neck junction lesions are often underrated in particular [9]. In this respect, mini-open arthrotomy provides direct vision to the pathomorphologies of the femoral heads and avoids excessive traction during the surgeries. Femoroacetabular clearance could be tested directly by placing the surgeon’s finger on acetabular margin while applying impingement maneuver to feel any squeeze from bony bump around the head-neck junction that is inadequately corrected. Impingement-free range of motion can be obtained while avoiding the need for hip surgical dislocation or prolonged excessive traction. The use of mini-open procedure also avoids inadvertent vascular injury in inexperienced hands while creating the anterior portal or during the arthroscopic procedure [35]. The technique also improves patient’s quality of life such as sleep disorders [36, 37].

**Table 5:** Comparison of studies on the outcomes of FAI surgery in non-athletic adults after 2007 and with a mean follow-up for at least 36 months

Author (year)	Surgery	No. of hips	FAI type	Mean follow-up (months)	Outcomes	THA or revision
Beaulé et al. (2007) [13]	SDH	37	Cam	37.2	WOMAC: 61.2 to 81.4	–
Graves et al. (2009) [11]	SDH	48	Cam and pincer	38	Merle d’Aubigné: 13 to 16.8	–
Naal et al. (2012) [25]	SDH	233	Cam and pincer	60.7	83% returned normal hip function	11.6%
Boone (2012) [30]	SDH	22	Cam and pincer	46.8	50% clinical failure	27%
Steppacher et al. (2015) [10]	SDH	93	Cam and pincer	131	Merle d’Aubigné: 15.3 to 16.9	11.8%
Peters et al. (2015) [1]	SDH	142	Cam and pincer	36	Risk factor: age and pre-op HHS	32%
Horisberger et al. (2010) [3]	Arthroscope	20	Cam and pincer	36	NASH: 47.15 to 78.3	40%
Palmer et al. (2012) [4]	Arthroscope	201	Cam	46	NASH: 56.1 to 78.2	13%
Malviya et al. (2012) [15]	Arthroscope	612	Cam and pincer	38.4	Quality of life scores improved 76.6% of patients, and 9% deteriorated	–
Larson et al. (2012) [31]	Arthroscope	94	Cam and pincer	42	HHS: 64.5 to 94.3 (labral re-fix) and 64.7 to 84.9 (labral excision)	8.5%
Skendzel et al. (2014) [32]	Arthroscope	466	Cam and pincer	73	Outcomes better in preserved joint space group than limited joint space group	25.1%
Hufeland et al. (2016) [33]	Arthroscope	44	Mainly cam	66.3	HHS: 67.2 to 86.4	11.4
Menge et al. (2017) [29]	Arthroscope	145	Cam and pincer	120	No influence on outcomes for labral debridement or repair	34%
Our study	Mini-open and arthroscope	39	Cam	44	HHS: 61.1 to 84.2	23%

*SDH* surgical dislocation of hip

The risk factors of failure in the treatment of FAI were investigated in many studies [14, 18, 19, 21, 29]. Our results were comparable to those reported in other literature, showing that younger patients and patients in the early stages of hip osteoarthritis (Tönnis 0 or Tönnis I) might benefit from surgical treatments [9, 19]. Boone et al. supposed that caution should be taken to perform surgical dislocation in patients over the age of 40 [30]; in our series using minimally invasive approach, patients younger than 55 still gained benefit from the surgery. It still remained controversial if labral tears itself and the types of management to a torn labrum would influence the surgical outcomes [1, 38]. Laude et al. observed no difference in clinical results between patients with labral refixation and those without [18]. They suspected that refixation of a damaged labrum was responsible for persistent pain and revision by debridement in eight patients in their study. The same viewpoints also came from other authors [21, 29]. In our series, we did not notice a significant influence of labral tear on clinical outcomes, and most of our patients underwent debridement or excision due to degenerative or unrepairable tear. Larger sample size is needed to further define the importance of labral integrity and management in FAI.

Our study has limitations. First, mixed-type impingement was the most common form of FAI. However, in this study, we only included patients with cam-type lesion. This was due to the technical limitation of using mini-open anterolateral approach to address a retroverted or overcovered acetabulum in our experience. Surgical dislocation remained the gold-standard to do delicate acetabular osteoplasty or periacetabular osteotomy [6, 12, 23]. That being said, cam-type lesion was common in young male, and our procedure took the advantages of short hospital stay and rapid recovery to normal activity for these patients. Second, the number of the patients included was small and there was no control group (e.g., open surgical dislocation or hip arthroscopy). However, our patient-selection and surgical indications were well-defined, and the clinical and radiographic data were recorded in detail. The significant improvement in hip function after the surgery rendered our results exceptionally representative of the surgical outcomes of mini-open approach in the treatment of cam-type FAI. Third, the posterior femoral and labral lesions might be difficult to approach with this technique despite of the fact that posterior lesions were relatively rare. Finally, we did not use fracture table but only use manual traction of the lower limb when doing the hip arthroscopy. In muscular patients or patients with soft tissue scarring, traction by fracture table should be advisable to improve the intra-articular accessibility.

## Conclusions

The use of mini-open and arthroscopic osteochondroplasty is an effective, technically straightforward and minimally invasive procedure. The technique could be a viable surgical option for symptomatic cam-type FAI patients to improve hip function and relieve symptoms of mechanical impingement with low complication rate.

## Abbreviations

AI: Acetabular index
AP: Anteroposterior
BMI: Body mass index
FAI: Femoroacetabular impingement
HHS: Harris hip score
LCE angle: Lateral center-edge angle
SDH: Surgical dislocation of hip
THA: Total hip arthroplasty
VAS: Visual analogue scale
