# Correction to: Treatment of cam-type femoroacetabular impingement using anterolateral mini-open and arthroscopic osteochondroplasty

**DOI:** 10.1186/s13018-019-1291-x

**Published:** 2019-08-08

**Authors:** Cheng-Ta Wu, Mohammed Mahameed, Po-Chun Lin, Yu-Der Lu, Feng-Chih Kuo, Mel S. Lee

**Affiliations:** 1 Department of Orthopaedic Surgery, Kaohsiung Chang Gung Memorial Hospital, Kaohsiung, Taiwan; 2 Chang Gung University, College of Medicine, 123, Ta Pei Road, Niao Sung District, Kaohsiung, Taiwan

**Correction to: J. Orthop Surg Res**

**https://doi.org/10.1186/s13018-019-1257-z**

In the original publication of this article [1], the first name of the 5th author is wrong.

The correct name should be Feng-Chih Kuo.

The original article has been corrected.
